# Migrant workers in Italy: an analysis of injury risk taking into account occupational characteristics and job tenure

**DOI:** 10.1186/s12889-017-4240-9

**Published:** 2017-04-22

**Authors:** Massimiliano Giraudo, Antonella Bena, Giuseppe Costa

**Affiliations:** 1 Department of Epidemiology, Servizio di Epidemiologia, ASL TO3, Via Sabaudia 164 - 10095 Grugliasco, Turin, Italy; 2 Department of Clinical and Biological Sciences, University of Turin, Orbassano, Turin, Italy

**Keywords:** Occupational injuries, Migrant workers, Job tenure

## Abstract

**Background:**

Migrants resident in Italy exceeded 5 million in 2015, representing 8.2% of the resident population. The study of the mechanisms that explain the differential health of migrant workers (as a whole and for specific nationalities) has been identified as a priority for research. The international literature has shown that migrant workers have a higher risk of total and fatal injury than natives, but some results are conflicting.

The aim of this paper is to study the injury risk differentials between migrants, born in countries with strong migratory pressure (SMPC), and workers born in high income countries (HIC), taking into account individual and firm characteristics and job tenure. In addition to a comprehensive analysis of occupational safety among migrants, the study focuses on Moroccans, the largest community in Italy in the years of the analysis.

**Methods:**

Using the Work History Italian Panel-Salute integrated database, only contracts of employment in the private sector, starting in the period between 2000 and 2005 and held by men, were selected. The analysis focused on economic sectors with an important foreign component: engineering, construction, wholesale and retail trade, transportation and storage. Injury rates were calculated using a definition of serious occupational injuries based on the type of injury. Incidence rate ratios (IRR) were calculated using a Poisson distribution for panel data taking into account time-dependent variables.

**Results:**

Injury rates among SMPC workers were higher than for HIC workers in engineering (15.61 ‰ py vs. 8.92 ‰ py), but there were no significant differences in construction (11.21 vs. 10.09), transportation and storage (7.82 vs. 7.23) and the wholesale and retail sectors (4.06 vs. 4.67). Injury rates for Moroccans were higher than for both HIC and total migrant workers in all economic sectors considered. The multivariate analysis revealed an interaction effect of job tenure among both SMPC and Moroccan workers in the construction sector, while in the wholesale and retail trade sector an interaction effect of job tenure was only observed among Moroccan workers.

**Conclusions:**

Migrant workers have higher occupational injury rates than Italians in the engineering and construction sectors, after two years of experience within the job. Generally the risk differentials vary depending on the nationality and economic sector considered. The analysis of injury risk among migrant workers should be restricted to serious injuries; furthermore, job tenure must be taken into account.

**Electronic supplementary material:**

The online version of this article (doi:10.1186/s12889-017-4240-9) contains supplementary material, which is available to authorized users.

## Background

Migrants resident in Italy exceeded 5 million in 2015, representing 8.2% of the resident population. This percentage has doubled since 2005, when it was 4.1% [1]. Since 2008, following the entry of Eastern European countries in the European Union, Romanians have represented the largest proportion of migrants, followed by Albanians and Moroccans. The main reason for immigration is economic opportunity: migrants are generally younger people, who represent a higher fraction of the active population than Italians [2]. There are about 2.3 million migrant workers in Italy (10%), mainly concentrated in Northern Italy (59%) [3]. In the Italian labour market the demand for migrant workers tends to be concentrated on one hand, on highly skilled work, and, on the other, on un-skilled and precarious work (the so-called 3D jobs: dirty, dangerous, and demanding) [4]. According to the Italian National Institute of Statistics (ISTAT), 91,000 foreign citizens have reported sustaining an occupational injury, with an incidence rate of 3.3%, which is higher than that reported among the Italian population (2.8%) [5]. The Italian Workers Compensation Authority (INAIL) has reported that 52,742 occupational injuries were sustained by migrant workers in 2014 (14% of the total of occupational injuries) and that, of these, 72 resulted in death (14.6%) [6]. The international literature has shown that migrant workers have a higher risk of total and fatal injury than natives, although some results are conflicting [7]. The main reasons are due to the segregation of migrants, who are assigned to the most dangerous jobs and required to perform the most dangerous tasks within these jobs, and to the transient nature of much of the work (migrants are constantly shifting between unemployment, underemployment and informal work) [8]. Other factors that have been identified are the different perception of job risk, linguistic barriers and cultural factors that reduce the effectiveness of any training [9].

The literature also indicates that in many highly developed countries, migrants are more frequently employed under fixed-term contracts, [10] and this finding has been confirmed in Italy [11]. The proportion of foreigners in precarious jobs is higher than that of Italian workers [12].

One of the consequences of flexibility is the fragmentation of the overall career path. Many studies have established that newly hired workers – whatever the type of employment contract – are more likely to suffer an injury than those with longer job tenures [13, 14].

A recent study has confirmed that the inverse association between job tenure and injury risks persists, despite the effect of confounding due to background variables and previous experience [15].

The study of these issues and the mechanisms that explain the differential health of migrant workers has been identified as a priority for research [16].

The literature has pointed out that the overall impact of migration on health is still not well known, also due to a lack in many countries of adequate occupational surveillance systems to monitor the health problems of migrant workers. Often official data do not permit injury risk assessments based on personal or employment characteristics. This limitation also applies to the Italian context. Each year the National Insurance Institute for Occupational Injuries (INAIL) reports the number of occupational injuries that occurred among migrant workers, but does not provide information about injury rates. This is due to the lack of suitable denominators. A study conducted by the National Institute of Statistics (ISTAT) has shown that work-related injuries were significantly higher among male migrant workers than Italian ones [17]. This study was based on a section (“Health and safety in the workplace”) that was only included in the LFS questionnaire for the 2nd quarter of 2007.

In order to monitor workers’ health over time, the Italian Ministry of Health has created a new occupational surveillance system (called WHIP-Salute), based on record linkage of administrative data, which contains information about occupational injuries and the workers involved [18]. Whip-salute contains useful information for studying safety among migrant workers, that is not currently available in the national data base.

The aim of this paper is to study the injury risk differentials between migrants and natives, taking into account individual (age) and firm (geographic area, size) characteristics, and job tenure. In addition to a comprehensive analysis of occupational safety among migrants, the study focuses on Moroccans, the largest community in Italy in the years of the analysis.

## Methods

### The WHIP-salute dataset

The Work and Health Histories Italian Panel (WHIP-Salute) is a database of individual work histories derived from the administrative archives of the National Institute for Social Welfare (INPS). The dataset currently covers the period from 1985 to 2005. The reference population (about 15 million individuals) is made up of people who have worked for the whole or part of their career in Italy. A systematic sample of 7% of this population was extracted on the basis of their date of birth. For every individual the career path was reconstructed: work periods, retirement, unemployment benefits, redundancy payments, disability indemnities. WHIP-Salute comprises employed work, non-professional self-employed work, and subcontracted work; some professional categories, such as architects and lawyers, are not included. It covers all production sectors in manufacturing, construction and services, but does not include permanent workers in the public sector and all workers in the agricultural sector. The database comprises a great variety of demographic and employment information. The section regarding employed work is a linked employer-employee database (LEED): thanks to the link with the INPS Observatory of enterprises, workers’ data are merged with information about the companies which employ them.

Using the same sampling criteria used for the INPS archives, episodes of occupational injury that occurred between 1994 and 2005 and resulted in more than three days’ absence from work, as certified by a physician, were extracted from the archive of the National Insurance Institute for Occupational Injuries (INAIL). A linkage between the two was then established using an encrypted unique identifier based on the worker’s tax code; this was performed separately by the two organisations. All activities, regardless of their complexity or depth, were conducted in accordance with Italian data protection regulations and with the approval of the national institutes involved.

From 2013, the WHIP-Salute database was included, under the responsibility of the Ministry of Health, in the National Statistics Program that establishes which statistical surveys are of public interest. Our institution participates in the database development program, so the data we used were freely available to us. In other cases, the Ministry of Health releases microdata files for research purposes, upon request based on a research protocol and after obtaining the prior authorization of the Italian Data Protection Authority. For a more detailed description of the WHIP-Salute database see Bena et al. [18].

The WHIP-Salute database is the result of the linkage of administrative data extracted from the archives managed. It is based on a systematic sampling of individuals on the basis of their day of birth. The hypothesis underlying this choice is that the probability of extracting an individual is uniform throughout the month. This assumption is not always valid in the case of migrant workers. Sometimes foreign workers born in selected countries do not know their exact date of birth. For this reason at the time of registration in the official records in Italy, they state that they were born on January 1. This generates an oversampling differential by country of birth. Oversampling is not necessarily a problem. In fact, if we wanted to study a particular nationality, oversampling would not generate bias. On the contrary, since the sampling fraction would be higher than the original one, we would obtain more accurate estimates. But when (as in this case) the aim is to compare occupational injury rates between migrant workers (as a whole) and natives, oversampling would generate bias. To correct this distortion, we used the Italian LFS to assign a weight to each worker, based on his country of birth.

### Definition of variables

The analysis only considered contracts of employment in the private sector starting between 2000 and 2005, held by men aged between 16 and 55 years hired as blue collar or apprentice workers. We focused on the economic sectors with a large foreign component (65% of person-years): engineering, construction, wholesale and retail trade, transportation and storage.

Two main groups were defined based on country of birth: high income countries (HIC: as defined by the World Bank; this group included Italians, who represented 98% of the group) and countries with strong migratory pressure (SMPC: Africa; Asia excluding Israel, South Korea and Japan; Latin America; Central and Eastern Europe). We also considered Moroccans, the largest community in Italy in those years.

All analyses were performed using only serious injuries to limit bias due to underreporting. For the present analysis we used a definition based on the type of injury: anatomical loss, foreign bodies, fracture (hand, wrist, arms, chest, cervical, thoracic, lumbar, sacral column, pelvis, hip, knee, ankle, foot) or fatal events. We did not use a definition based on time off work, often used as a proxy for injury severity, since instances of early return to work by foreign workers and underreporting of events with a long prognosis have been reported in Italy [19].

Time at risk was calculated on the basis of months actually worked, subtracting all periods of absence from work due to illness or injury and temporary lay-off from paid months.

Job tenure is a time-dependent variable calculated as the time since the beginning of the contract. Its maximum length was 72 months.

### Statistical analysis

Injury rates per 1000 person-years were calculated (with 95% confidence intervals – 95% CI) for country of birth, stratified by economic sectors and job tenure.

Multivariate analysis was performed in order to calculate incidence rate ratios (IRR) for country of birth, stratified by economic sectors, using a Poisson distribution for panel data that takes into account time-dependent variables. The 95% CI were calculated applying the correction for repeated events [20].

We calculated three models:

- model 1: unadjusted;
- model 2: adjusted for background variables: age (linear), firm size (0–9, 10–19, 20–199, >199), firm geographic area (Northwest, Northeast, Central, South and Islands), years of work;
- model 3: model 2 plus job tenure (linear).

The analyses were performed using SAS 9.2 and Stata 13.

## Results

812,624 person-years were considered during the study period, equal to 397,986 workers (see Additional file 1).

Migrants had shorter job tenures than Italians in all the economic sectors considered (Table 1): the difference in the proportion of person-years with a job tenure of less than 1 year ranged between 3% (engineering) and 10% (transportation and storage). The same results applied to Moroccans.

**Table 1:** Number of occupational injuries and person-years stratified by country of birth, economic sector and job tenure

Economic sector	Job tenure (in months**)**	HIC			SMPC			of which born in Morocco		
		Injuries	Person-years	%	Injuries	Person-years	%	Injuries	Person-years	%
Engineering										
	< 6	289	25,557	21.2	201	11,060	23.1	101	4208	23.2
	6–12	239	25,542	21.2	202	10,896	22.8	81	4029	22.2
	13–24	254	29,671	24.7	184	11,725	24.5	77	4354	24
	> 24	292	39,526	32.9	202	14,191	29.6	93	5559	30.6
	Total	1074	120,296	100	789	47,872	100	352	18,149	100
Construction										
	< 6	517	41,370	30.1	270	20,193	33.8	126	8622	34.6
	6–12	339	33,893	24.7	146	16,142	27	71	6585	26.4
	13–24	284	31,759	23.1	164	13,246	22.2	90	5351	21.5
	> 24	249	30,311	22.1	130	10,144	17	71	4355	17.5
	Total	1389	137,333	100	710	59,725	100	358	24,912	100
Wholesale and retail trade										
	< 6	108	17,343	22.6	23	3510	27	9	1258	28.2
	6–12	94	17,406	22.7	15	3202	24.7	7	1072	24
	13–24	78	19,596	25.6	21	3155	24.3	18	1043	23.4
	> 24	78	22,326	29.1	20	3110	24	11	1086	24.4
	Total	358	76,670	100	79	12,977	100	45	4458	100
Transportation and storage										
	< 6	126	12,709	27.3	68	6378	34.1	31	2818	36.1
	6–12	79	10,775	23.2	43	4948	26.5	16	2050	26.3
	13–24	67	10,899	23.4	32	4181	22.4	21	1683	21.6
	> 24	65	12,122	26.1	21	3189	17.1	14	1249	16
	Total	337	46,505	100	164	18,696	100	82	7801	100
Other sectors										
	< 6	399	57,551	28.9	233	29,240	31.4	120	9814	30.5
	6–12	268	43,897	22	147	22,813	24.5	73	7624	23.7
	13–24	219	44,687	22.4	119	20,649	22.2	64	7105	22.1
	> 24	226	53,231	26.7	118	20,483	22	59	7666	23.8
	Total	1112	199,365	100	617	93,184	100	316	32,209	100
Total										
	< 6	1439	154,531	26.6	795	70,380	30.3	387	26,720	30.5
	6–12	1019	131,513	22.7	553	58,001	25	248	21,360	24.4
	13–24	902	136,611	23.5	520	52,955	22.8	270	19,536	22.3
	> 24	910	157,515	27.1	491	51,117	22	248	19,913	22.8
	Total	4270	580,170	100	2359	232,453	100	1153	87,529	100

6629 injuries occurred between 2000 and 2005.

Table 2 shows injury rates by country of birth and job tenure, stratified by four main economic sectors (520,074 person-years). Injury rates among SMPC workers were higher than among HIC workers in the engineering sector (15.61‰ person-years vs. 8.92‰ person-years), but no significant differences were observed in construction (11.21 vs. 10.09), transportation and storage (7.82 vs. 7.23) or wholesale and retail sectors (4.06 vs. 4.67). Compared to both HIC and total migrant workers, Moroccans showed higher injury rates in all the economic sectors considered.

**Table 2:** Crude injury rates per 1000 workers by country of birth, economic sector and job tenure

Economic sector	Job tenure (in months)	HIC		SMPC		of which born in Morocco	
		Injury rate (per 1000 person-years)	CI 95%	Injury rate (per 1000 person-years)	CI 95%	Injury rate (per 1000 person-years)	CI 95%
Engineering							
	< 6	11.27	9.97–12.57	17.61	13.73–21.50	24.00	19.32–28.68
	6–12	9.36	8.17–10.54	16.19	12.45–19.93	19.86	15.51–24.21
	13–24	8.56	7.51–9.61	16.03	12.43–19.64	17.69	13.74–21.64
	> 24	7.39	6.54–8.23	13.15	10.11–16.19	16.73	13.33–20.13
	Total	8.91	8.37–9.44	15.61	13.84–17.38	19.34	17.32–21.36
Construction							
	< 6	12.4	11.33–13.47	13.32	10.97–15.66	14.5	11.96–17.04
	6–12	10	8.94–11.07	8.6	6.52–10.67	10.78	8.27–13.29
	13–24	8.94	7.90–9.98	10.78	8.24–13.33	16.82	13.34–20.29
	> 24	8.21	7.19–9.24	11.86	8.78–14.94	16.3	12.51–20.10
	Total	10.09	9.56–10.62	11.21	9.97–12.44	14.33	12.84–15.82
Wholesale and retail trade							
	< 6	6.23	5.05–7.40	5.87	2.03–9.70	6.36	1.95–10.77
	6–12	5.4	4.31–6.49	4.11	0.82–7.40	6.53	1.69–11.37
	13–24	3.98	3.10–4.86	0.68	0.00–2.02	17.26	9.29–25.24
	> 24	3.49	2.72–4.27	5.53	1.70–9.37	10.13	4.14–16.12
	Total	4.67	4.19–5.15	4.06	2.44–5.69	9.87	6.95–12.78
Transportation and storage							
	< 6	9.84	8.11–11.56	12.23	7.93–16.54	10.64	6.84–14.45
	6–12	7.33	5.71–8.95	6.32	2.89–9.76	7.8	3.98–11.63
	13–24	6.15	4.68–7.62	6.11	2.50–9.72	12.48	7.14–17.81
	> 24	5.36	4.06–6.67	4.27	0.85–7.68	11.21	5.34–17.09
	Total	7.23	6.46–8.00	7.82	5.86–9.79	10.38	8.12–12.64

Injury rates among HIC workers decreased as job tenure increased; the same trend was observed in each economic sector considered. Among SMPC and Moroccan workers this trend was only observed in engineering, whereas no differences emerged in the other three sectors considered.

Table 3 shows the results of the multivariate analysis.

**Table 3:** Incidence rate ratio by country of birth, stratified by economic sectors and adjusted for background variables and job tenure

Economic sector	Country of birth	Model 1		Model 2		Model 3	
		IRR	CI 95%	IRR	CI 95%	IRR	CI 95%
Engineering							
	HIC	1	-	1	-	1	-
	SMPC	1.80	1.60–2.03	1.80	1.59–2.04	1.77	1.56–2.01
	of which Morocco	2.17	1.91–2.46	2.15	1.88–2.46	2.13	1.86–2.43
Construction							
	HIC	1	-	1	-	1	-
	SMPC	1.08	0.97–1.21	1.10	0.98–1.23	Interaction by job tenure^a^	
	of which Morocco	1.42	1.26–1.60	1.41	1.24–1.60	Interaction by job tenure^a^	
Wholesale and retail trade							
	HIC	1	-	1	-	1	-
	SMPC	0.94	0.68–1.31	0.96	0.69–1.34	0.91	0.65–1.27
	of which Morocco	2.11	1.55–2.88	2.07	1.51–2.83	Interaction by job tenure^b^	
Transportation and storage							
	HIC	1	-	1	-	1	-
	SMPC	1.06	0.85–1.33	1.02	0.81–1.30	0.98	0.77–1.25
	of which Morocco	1.43	1.12–1.83	1.31	1.00–1.70	1.27	0.97–1.66

model 1: unadjusted;

model 2: adjusted for background variables: age (as a continuous variable), firm geographic area (based on Italy’s administrative boundaries), firm size (yearly average number of employees), years of work

model 3: model 2 plus job tenure (as a continuous variable)

^a^IRRs stratified by job tenure presented in Table 4

^b^IRRs stratified by job tenure presented in Table 5

Compared to the crude IRR, the estimate in the final model did not change in the engineering sector (1.77, 95% CI 1.56–2.01 vs. 1.80, 95% CI 1.60–2.03). The risk was greater among Moroccans than SMPC workers (model 3 estimate: 2.13, 95% CI 1.86–2.43).

Compared to HIC workers, SMPC construction workers did not have a significant differential risk, either in the unadjusted model (IRR: 1.08, 95% CI 0.97–1.21) or in the second one (IRR: 1.10, 95% CI 0.98–1.23). The final model (Table 4) revealed an effect modification by job tenure: the IRR among SMPC workers increased with job tenure. After 2 years from the start of the contract, the risk for foreign workers was 1.16 (95% CI 1.01–1.34), after six years it was 1.68 (95% CI 1.03–2.75). Interaction by job tenure was also observed among Moroccans, for whom the risk was always higher when compared to SMPC. Compared to HIC workers, the IRR was significantly higher at the beginning of the employment contract (IRR: 1.19, 95% CI 1.01–1.40), and increased strongly: after 2 years the IRR was 1.56 (95% CI 1.33–1.82), after 6 years it was 2.72 (95% CI 1.63–4.54).

**Table 4:** Incidence rate ratio by country of birth and job tenure, adjusted for individual and occupational variables; construction sector

Job tenure (in months)	HIC	SMPC		of which born in Morocco	
	IRR	IRR	CI 95%	IRR	CI 95%
1	1.00	0.97	0.84–1.14	1.19	1.01–1.40
6	1.00	1.01	0.89–1.15	1.26	1.10–1.45
12	1.00	1.06	0.94–1.19	1.35	1.19–1.54
18	1.00	1.11	0.98–1.25	1.45	1.27–1.66
24	1.00	1.16	1.01–1.34	1.56	1.33–1.82
36	1.00	1.28	1.03–1.58	1.79	1.42–2.25
48	1.00	1.40	1.03–1.89	2.06	1.49–2.83
60	1.00	1.53	1.03–2.28	2.36	1.56–3.58
72	1.00	1.68	1.03–2.75	2.72	1.63–4.54

In the wholesale and retail trade sector no significant risk differences were observed among SMPC workers compared to HIC workers. Moroccans showed an interaction by job tenure (Table 5): the IRR increased with job tenure. Compared to HIC workers, the risk after 6 months was 52% higher among Moroccans (95% CI 1.04–2.24). After 2 years, the risk was more than twice as high (IRR: 2.46, 95% CI 1.76–3.42); after six years the risk was 8 times higher (IRR: 8.79, 95% CI 3.35–23.11).

In the transportation and storage sector, after controlling for occupational characteristics and job tenure, there were no differences in the estimated risk. Compared to HIC, SMPC workers (IRR: 0.98, 95% CI 0.77–1.25) had a similar risk. However, considering Moroccans only, the risk was higher (IRR: 1.27, 95% CI 0.97–1.55).

**Table 5:** Incidence rate ratio among Moroccans by job tenure, adjusted for individual and occupational variables; wholesale and retail trade sector

Job tenure (in months)	HIC	Born in Morocco	
	IRR	IRR	CI 95%
1	1.00	1.33	0.86–2.07
6	1.00	1.52	1.04–2.24
12	1.00	1.79	1.27–2.50
18	1.00	2.09	1.52–2.88
24	1.00	2.46	1.76–3.42
36	1.00	3.38	2.19–5.22
48	1.00	4.65	2.56–8.42
60	1.00	6.39	2.94–13.88
72	1.00	8.79	3.35–23.11

## Discussion

Occupational injury rates among migrant workers were not generally higher than among natives, but varied depending on nationality, economic sector and job tenure considered. Our analysis showed that occupational injury rates were higher among migrant workers than Italians in the engineering and construction sectors, after two years of experience within the job. Moroccan workers were found to have higher injury rates, regardless of the economic sector, compared to both HIC workers, and migrant workers as a whole.

The results revealed four main findings.

First, it is important to take into account the specificity of each nationality in order to avoid bias in the results. As a general rule, epidemiological studies on immigrants’ health stratify the analysis by nationality. Instead, studies on occupational injury consider immigrants as a homogeneous group of individuals: this is a mistake.

Secondly, when analyzing occupational injuries among migrant workers it is important to consider the role of job tenure. Job tenure is an important determinant of the risk of occupational injury. Newly hired workers, whatever the type of contract, have a higher risk of occupational injury than individuals with a longer job tenure [13–15]. In the current period, in which the labour market is increasingly flexible and job switches are common, workers more and more frequently find themselves in a period of “high risk”. This precarious employment status is particularly frequent among migrant workers [11, 12]. For these reasons it is important to consider job tenure as a possible confounding factor. Our results (Table 3) show that job tenure has different effects on the analysis of exposure by migrant workers to injury risk, according to the economic sector considered. In the engineering and transportation and storage sectors, even controlling for job tenure, the risk of work injury was found to be substantially unchanged, for both SMPC and Moroccan workers.

In the construction sector (Table 4) an interaction effect of job tenure was observed for both SMPC and Moroccan workers, while in the wholesale and retail trade sector there was only an interaction effect of job tenure for Moroccan workers (Table 5). The risk of occupational injury increased with the duration of job tenure. This mechanism is explained by the fact that occupational injury rates among natives decrease as job tenure increases. This finding is consistent with the epidemiological literature on these issues [14]. Injury rates among SMPC workers in the construction sector and Moroccan workers in the construction and wholesale and retail trade sectors did not decrease. One possible explanation is that the injury rate among HIC workers decreases as they acquire experience and familiarity with the work organization, also through interaction with colleagues. Furthermore, as their job tenure increases, HIC workers can negotiate the type of job they perform, and are involved in simpler and less dangerous tasks [21]. Migrant workers may find it more difficult to integrate with colleagues, and to adapt to the work organization, because of their poorer language skills and cultural barriers. At the same time, SMPC workers are probably less likely than HIC workers to be able to negotiate the type of tasks they perform, and, at the same time, they might have less information about the risks associated with the job, due to the lower levels of social capital [22].

SMPC workers might be more willing than HIC workers to perform tasks with higher risks, because they are afraid of losing their jobs [23]. Demand for job amenities, including workplace safety, increases as wealth increases. Migrants usually have lower incomes than natives and probably tend to trade off higher wages for worse conditions [24]. Furthermore, inadequate training, due to communication difficulties and low investment by employers, results in failure to assimilate the contents and adopt appropriate behaviour [25].

The third finding is the evidence that in the analysis of occupational injury risk among migrant workers, the sample must be stratified by economic sector, since the composition by nationality differs according to the sector. In fact, the analysis revealed that both migrant workers and Moroccans have different injury risks in the economic sectors considered (see Table 3). There are sectors (e.g., engineering) where the most dangerous tasks are systematically assigned to migrant workers of any nationality. This does not happen in other sectors. A study conducted among workers engaged in the construction of a high speed railway showed that workers were employed in different ways depending on nationality, with North African workers assigned to lower level tasks than Italian and other nationalities (e.g., Romanian) [26].

The last suggestion is that, to control the phenomenon of underreporting, all analyses of occupational injuries among foreign workers should be restricted to serious injuries.

Numerous studies have indicated that underreporting is particularly frequent among migrant workers because they might be concerned about possible reprisal and might not be able to afford time away from work [19, 27]. By selecting injuries according to type, it is possible to identify events that, owing to their characteristics, cannot be ignored (and therefore not be reported).

The main limits are related to our sample selection. Our study was restricted to male workers, employed as blue collar workers or apprentices: the results for this category of worker are reliable but cannot be extended to women and white-collar workers. Among temporary contracts we only selected fixed-term contracts, seasonal work and on-the-job training contracts. Jobs obtained through temporary work agencies were excluded, because in this case the INPS archives do not include the data necessary to take into account confounder effects (i.e., economic sector, firm size at the time of the injury). The present paper does not include self-employed workers. The number of individual companies owned by foreign nationals has increased in recent years [28]. This is not always a free choice, but may be requested by employers: without an employment contract, employers are not required to train or insure workers, and are not obliged to provide the necessary work equipment. In addition, employers assign the most dangerous tasks to such self-employed workers.

WHIP-Salute only contains information about workers registered with the INPS: it does not consider the entire immigrant population in Italy, which also includes a proportion of illegal workers and undocumented migrants, reported to be at high risk of accidents [19]. The results for this sample of workers are quite reliable but, in order to test whether our conclusions can be generalised to the entire Italian population, further analyses must be conducted on female and self-employer.

## Conclusions

Our study attempts to respond to the needs of research and the challenges that the study of this issue poses [16]. Most countries do not have adequate national systems to monitor key occupational health problems among migrants and most official and unofficial statistics do not disaggregate migratory flows by age, gender, ethnicity, or social class.

WHIP-Salute allowed us to analyze high quality data, and obtain highly detailed information about employment characteristics. Furthermore, the main characteristics of WHIP-Salute – its national representativeness and longitudinal nature - allow the assessment of the health of migrant workers in years of important transformation of the labour market.

Our study examines the safety of migrant workers in the years of economic growth, where the employment rate increased, and the unemployment rate decreased [3]. This allowed us to assess the conditions of migrant workers in a favourable economic context. The economic crisis that began in 2008 has mainly affected the working conditions of migrants.

## Additional file

Additional file 1: Main characteristics of the workers considered in the study. (DOC 91 kb)

## Abbreviations

CI: Confidence interval
HIC: Highly developed countries as defined by the World Bank
INAIL: Italian workers compensation authority
INPS: Italian National Social Security Institute
IRR: Incidence rate ratio
ISTAT: Italian National Institute of Statistics
LEED: Linked employer-employee database
SMPC: Countries with strong migratory pressure
WHIP-Salute: Work and Health Histories Italian Panel
