# Supplementary material for: Migrant workers in Italy: an analysis of injury risk taking into account occupational characteristics and job tenure
Source: BMC Public Health. 2017 Apr 22;17:351. doi: 10.1186/s12889-017-4240-9 (PMC5401389; doi:10.1186/s12889-017-4240-9)
Supplement: Additional file 1: — Main characteristics of the workers considered in the study. (DOC 91 kb) [file 12889_2017_4240_MOESM1_ESM.doc]

# ADDITIONAL FILE 1

**Main characteristics of the workers considered in the study**

|  | | PSA | | | PFPM | | | of which born in | | |
| --- | --- | --- | --- | --- | --- | --- | --- | --- | --- | --- |
|  |  |  |  |  |  |  |  | Morocco | | |
|  |  | Num. Injuries | Person years | % | Num. Injuries | Person years | % | Num. Injuries | Person years | % |
| **Economic sector** | | | | | | | | | | |
|  | Engineering | 1,074 | 120,296 | 20.73 | 789 | 47,883 | 20.59 | 352 | 18,149 | 20.73 |
|  | Construction | 1,389 | 137,333 | 23.67 | 710 | 59,727 | 25.69 | 358 | 24,912 | 28.46 |
|  | Wholesale and retail trade | 358 | 76,670 | 13.22 | 80 | 12,988 | 5.59 | 45 | 4,458 | 5.09 |
|  | Transportation and storage | 337 | 46,505 | 8.02 | 164 | 18,696 | 8.04 | 82 | 7,801 | 8.91 |
|  | Other sectors | 1,112 | 199,365 | 34.36 | 617 | 93,226 | 40.09 | 316 | 32,209 | 36.80 |
| **Job tenure (in months)** | | | | | | | | | | |
|  | < 6 | 1,439 | 154,531 | 26.64 | 795 | 70,403 | 30.28 | 387 | 26,720 | 30.53 |
|  | 6 - 12 | 1,019 | 131,513 | 22.67 | 554 | 58,017 | 24.95 | 248 | 21,360 | 24.40 |
|  | 13 - 24 | 902 | 136,611 | 23.55 | 520 | 52,971 | 22.78 | 270 | 19,536 | 22.32 |
|  | > 24 | 910 | 157,515 | 27.15 | 491 | 51,128 | 21.99 | 248 | 19,913 | 22.75 |
| **Age** | | | | | | | | | | |
|  | 16 - 24 | 988 | 141,825 | 24.45 | 271 | 30,100 | 12.94 | 115 | 9,580 | 10.94 |
|  | 25 - 34 | 1,420 | 205,520 | 35.42 | 955 | 97,987 | 42.14 | 439 | 34,968 | 39.95 |
|  | 35 - 44 | 1,074 | 141,581 | 24.40 | 803 | 75,974 | 32.67 | 418 | 30,333 | 34.66 |
|  | 45 - 55 | 788 | 91,244 | 15.73 | 331 | 28,459 | 12.24 | 181 | 12,647 | 14.45 |
| **Firm geographic area** | | | | | | | | | | |
|  | Northwest | 1,154 | 154,068 | 26.56 | 977 | 93,914 | 40.39 | 537 | 40,195 | 45.92 |
|  | Northest | 988 | 126,500 | 21.80 | 927 | 85,160 | 36.62 | 427 | 31,680 | 36.19 |
|  | Central | 712 | 104,350 | 17.99 | 376 | 41,073 | 17.66 | 163 | 12,002 | 13.71 |
|  | South and Islands | 1,416 | 195,252 | 33.65 | 80 | 12,374 | 5.32 | 26 | 3,651 | 4.17 |
| **Firm size (yearly average number of employees)** | | | | | | | | | | |
|  | 0 - 9 | 1,602 | 232,452 | 40.07 | 977 | 91,495 | 39.35 | 537 | 33,121 | 37.84 |
|  | 10-19 | 776 | 99,132 | 17.09 | 927 | 40,937 | 17.61 | 427 | 15,199 | 17.36 |
|  | 20 - 199 | 1,322 | 167,778 | 28.92 | 376 | 73,139 | 31.45 | 163 | 29,480 | 33.68 |
|  | > 199 | 570 | 80,809 | 13.93 | 80 | 26,949 | 11.59 | 26 | 9,729 | 11.12 |
| **Year of work** | | | | | | | | | | |
|  | 2000 | 335 | 37,477 | 6.46 | 159 | 11,675 | 5.02 | 85 | 4,993 | 5.70 |
|  | 2001 | 659 | 75,197 | 12.96 | 290 | 24,584 | 10.57 | 144 | 10,173 | 11.62 |
|  | 2002 | 757 | 98,792 | 17.03 | 383 | 36,997 | 15.91 | 192 | 14,628 | 16.71 |
|  | 2003 | 825 | 112,916 | 19.46 | 462 | 48,896 | 21.03 | 218 | 17,986 | 20.55 |
|  | 2004 | 865 | 122,518 | 21.12 | 549 | 53,047 | 22.81 | 272 | 19,233 | 21.97 |
|  | 2005 | 829 | 133,269 | 22.97 | 517 | 57,322 | 24.65 | 242 | 20,515 | 23.44 |
| **Total** | | | | | | | | | | |
|  | | 4,270 | 580,170 | 100 | 2,360 | 232,520 | 100 | 1,153 | 87,529 | 100 |
